# A Novel Spore Wall Protein from *Antonospora locustae* (Microsporidia: Nosematidae) Contributes to Sporulation

**DOI:** 10.1111/jeu.12410

**Published:** 2017-04-10

**Authors:** Longxin Chen, Runting Li, Yinwei You, Kun Zhang, Long Zhang

**Affiliations:** ^1^ Department of Entomology China Agricultural University Beijing 100193 China; ^2^ Molecular Biology Laboratory Zhengzhou Normal University Zhengzhou 450044 China; ^3^ Bio‐tech Research Center Shandong Academy of Agricultural Sciences Jinan 250100 China

**Keywords:** Locust, RNAi, SWP

## Abstract

Microsporidia are obligate intracellular parasites, existing in a wide variety of animal hosts. Here, we reported *Aloc* SWP2, a novel protein identified from the spore wall of *Antonospora locustae* (formerly, *Nosema locustae*, and synonym, *Paranosema locustae*), containing four cysteines that are conserved among the homologues of several Microspodian pathogens in insects and mammals. *Aloc* SWP2 was detected in the wall of mature spores via indirect immunofluorescence assay. In addition, immunocytochemistry localization experiments showed that the protein was observed in the wall of sporoblasts, sporonts, and meronts during sporulation within the host body, also in the wall of mature spores. *AlocSWP2* was not detected in the fat body of infected locust until the 9th day after inoculating spores via RT‐PCR experiments. Furthermore, the survival percentage of infected locusts injected with dsRNA of *AlocSWP*2 on the 15th, 16th, and 17th days after inoculation with microsporidian were significantly higher than those of infected locusts without dsRNA treatment. Conversely, the amount of spores in locusts infected with *A. locustae* after treated with RNAi *AlocSWP2* was significantly lower than those of infected locusts without RNAi of this gene. This novel spore wall protein from *A. locustae* may be involved in sporulation, thus contributing to host mortality.

MICROSPORIDIA are intracellular parasites of all major Insecta and Mammalia classes and have a described diversity of over 1,400 species, although their actual diversity is estimated to be much higher (Szumowski and Troemel 2015; Williams 2009). Identification of the molecular mechanisms for pathogenicity of Microsporidia to its hosts is increasing remarkably with the progress of microsporidian genome sequencing. Although Microsporidia differ greatly in host range and cell type specificity, they share a similar mechanism for host cell invasion (Franzen 2005; Franzen et al. 2005). Infection involves the rapid expulsion of a polar tube from a dormant spore that pierces the host cell membrane and allows the direct transfer of the spore contents into the host cell cytoplasm (Williams 2009). A general viewpoint is that the spore wall is involved not only in this initial process, but also plays central roles in the complex interactions between Microsporidia and its host cells: including adherence, invasion, infection, and pathogenicity. Adherence of the microsporidian spores to host cells is the first step in the infection process (Southern et al. 2007) that occurs when spore wall proteins bind to sulfated glycosaminoglycans (GAGs) on the host cell surface (Hayman et al. 2005).

The microsporidian spore wall proteins identified so far, have very low or no sequence similarity with any other eukaryotic proteins: SWP1, SWP2, and EnP1 from *Encephalitozoon intestinalis* (Hayman et al. 2001; Southern et al. 2007); EnP1 from *Encephalitozoon romaleae* (Pombert et al. 2012); EcSWP1, EcCDA, EnP1, EnP2, and SWP3 from *Encephalitozoon cuniculi* (Brosson et al. 2005; Peuvel‐Fanget et al. 2006; Taupin et al. 2006; Xu et al. 2006); Ehswp1a and Ehswp1b from *Encephalitozoon hellem* (Polonais et al. 2010); as well as NbSWP7, NbSWP9, NbSW16, NbHSWP11, SWP5, NbSWP12, SWP25, SWP26, SWP30, and SWP32 from *Nosema bombycis* (Chen et al. 2013; Li et al. 2009, 2012; Wang et al. 2015; Wu et al. 2008, 2009; Yang et al. 2014, 2015, 2017). All of these spore wall proteins can be localized to the exospore, endospore, or plasmalemma, and nearly all of these have functional binding sites similar to the heparin‐binding motif (HBM), or modifications such as phosphorylation and glycosylation.

*Antonospora locustae* is an important pathogen, which has been commercialized and widely used for locust and grasshopper control (Brooks 1988; Henry 1971). Based on both molecular and morphological evidence, a change in the generic name of *Nosema locustae* to the genus *Antonospora* (*Paranosema*), as *A. locustae* n. comb. has been proposed (Slamovits et al. 2004; Sokolova et al. 2003). In particular, Microsporidia‐specific proteins such as spore wall proteins and polar tube proteins have received further attention (Dolgikh et al. 2005; Polonais et al. 2013). The close relative of *A. locustae*,* Paranosema grylli* was identified to have one spore‐wall protein via selective extraction of a major 40 kDa protein (Dolgikh et al. 2005). The spore‐wall and polar‐tube proteins are transported from the endoplasmic reticulum to the target membranes through these tubular networks (Beznoussenko et al. 2007). In aspects of host‐parasite interactions, the localization of hexokinase secreted by *A. locustae* into infected host cells suggests that some of Microsporidia possess a broad set of enzymes and regulatory proteins that have the potential to alter metabolic processes and molecular programs of the host (Senderskiy et al. 2014; Timofeev et al. 2016). However, little is known about the molecular pathogenicity of *A. locustae*, a potentially intriguing model system for understanding the extremes of reductive parasite evolution and host cell manipulation (Williams 2009).

In this paper, we have identified a putative spore wall protein of *A. locustae* via MALDI‐TOF mass spectrometry. Indirect immunofluorescence and immunochemistry localization experiments showed that this protein was localized in the spore wall. Furthermore, RNAi treatment against *AlocSWP*2 indicated that this protein was involved in sporulation, thus contributing to host mortality.

## Materials and Methods

### Microsporida and insects

*Antonospora locustae* spores were provided by the Kay Lab for Biocontrol of the Ministry of Agriculture of China, China Agricultural University, and were purified from its host locust (*Locusta migratoria*) in the laboratory. *Antonospora locustae* spores were purified from infected locust abdomens on a discontinuous Percoll gradient (25%, 50%, 75%, and 100%, v/v) centrifuged at 14,000 *g* for 20 min. Then, spores were washed with ultrapure water at least thrice. The purified spores were stored at −20 °C until further use (Gatehouse and Malone 1998).

Locusts were raised in our department at 28–30 °C, a relative humidity of 60%, and a photoperiod of 18:6 h light:dark. Fresh corn leaves were provided daily. To infect the locusts, locusts at 2nd or 3rd day of the 3rd instar were selected and starved for 4 h, then fed with 10^7^ purified spores of *A. locustae* on 20 mm × 5 mm corn leaves, then reared identical to healthy controls. The leaves should be completely consumed within 12 h, and locusts, which failed to do so, were not used in the vexperiment.

### Protein extraction, gel electrophoresis, and MALDI‐TOF MS assay

A small amount of the poorly soluble fraction of protein from *A. locustae* was extracted as follows. Briefly, using the Brosson method (Brosson et al. 2006), spores were disrupted in 200 μl of SDS extraction buffer, containing 100 mM DTT, 4% CHAPS and 0.2% SDS, by repeated cycles of freezing‐thawing and sonication (Scientz‐IIE, 300 W, 20–25 kHz) (“SDS extract”). The proteins from broken cells were extracted with a solution containing 7 M urea, 2 M thiourea, 100 mM DTT, 4% CHAPS, and 0.2% SDS for 6 h at room temperature. After clarification via centrifugation (12,000 *g*, 5 min), the supernatant or “urea extract” was collected and the sediment material treated with 30 mM NaOH overnight at 47 °C before centrifugation (as before) to collect the “NaOH extract” supernatant. The NaOH‐insoluble material, containing a small amount of spore proteins, was finally boiled for 10 min in Laemmli solution containing 2.5% SDS, 0.125 M Tris‐HCl pH 6.8, 20% glycerol, 2 mM EDTA, and 100 mM DTT (“Laemmli extract”).

Two‐dimensional electrophoresis (2‐DE) analysis was done as follows. Isoelectric focusing (IEF) was performed using linear immobilized pH gradient strips of 17 cm pH 3–10 (Bio‐Rad, Berkeley, CA) in rehydration buffer (7 M urea, 2 M thiourea, 4% CHAPS, 2 mM tributylphosphine solution, and 0.5% ampholytes) using the IPGphor apparatus (GE). After SDS‐PAGE on 12% polyacrylamide gels (18*18 cm), the strips were equilibrated with 50 mM Tris HCl pH 8.8, 6 M urea, 30% glycerol, 2% SDS, and 100 mM DTT, and then 135 mM iodoacetamide. After 2‐DE separation, the protein spots were manually excised from Coomassie brilliant blue (CBB) stained 2‐DE gels and transferred to a tube. The 2‐DE gel analysis was performed using the PDQuest 6.2.1 software. The protein spot in the gel was excised and digested by trypsin and submitted to for commercial MALDI‐TOF MS peptide mass finger printing analysis (Wu et al. 2008).

### 5′, 3′ rapid amplification of cDNA ends analysis of full‐length gene

Based on the peptides sequences identified by MALDI‐TOF MS analysis, RACE‐PCR primers were designed amplify the putative full‐length *AlocSWP2* gene, and the gene amplified by 5′ and 3′ RACE PCR with the SMARTer RACE Amplification kit (Clontech, 634859; Mountain View, CA), according to the manufacturer's protocol. Total RNA was extracted from spores using TRIzol (Invitrogen, 15596026; Waltham, MA). 5′‐RACE‐Ready cDNA and 3′‐RACE‐Ready cDNA were Synthesized using a 5′‐CDS Primer or a 3′‐CDS Primer A (included in the kit) respectively. The resulting cDNA was used as a template for PCR using an UPM (universal primer A mix) with the 5′ or 3′ *AlocSWP2*‐specific primers (5′‐RACE GSP: 5′‐AAYTTNGCYTCYTCNGTYTCNAGAAA‐3′, 3′‐RACE GSP: 5′‐ATGTTRAAYAAYTTYAAYAGRGATGA‐3′). Sequencing of the RACE‐PCR products thus confirmed amplification of the full‐length *AlocSWP2* gene.

### In silico analysis

Signal peptides were predicted by SignalP 4.1 Server (http://www.cbs.dtu.dk/services/SignalP/). Other modifications, such as N‐ and O‐glycosylation potential sites were predicted by NetOglyc (http://www.cbs.dtu.dk/services/NetOGlyc/) and NetNglyc (http://www.cbs.dtu.dk/services/NetNGlyc/) servers. Phosphorylation site prediction was also performed through the website, http://www.dabi.temple.edu/disphos/pred/predict. The search for glycosylphosphatidylinositol (GPI)‐anchorage was done using the DGPI algorithm (http://mendel.imp.ac.at/sat/gpi/gpi_server.html), and also by UniProt (http://www.uniprot.org/) and InterProScan (http://www.ebi.ac.uk/interpro/scan.html).

### Recombinant protein expression, purification, antibody production, SDS‐PAGE identification and Western analysis

The gene encoding *AlocSWP2* without signal coding sequence was amplified, via PCR or reverse transcription‐PCR (RT‐PCR), from *A. locustae* genomic DNA or total mRNA from infected locust by oligonucleotide primers *Aloc*SWP2‐F (5′‐CGGGATCCATCAGAACGGCAGCGACA‐3′), containing a *Bam*HI restriction site (underlined), and *Aloc*SWP2‐R (5′‐CGGAATTCTTAAGCAGAGTAGAAGCAGCG‐3′), containing an *Eco*RI restriction site (underlined) to facilitate cloning, were designed based on the predicted open reading frame KX255658. The amplified fragments were digested by *Bam*HI and *Eco*RI and cloned into the corresponding restriction enzyme digested expression vector pGEX‐4T‐2. The recombinant plasmid was transformed into *Escherichia coli* BL21 (DE3). After induction of expression by IPTG (0.5 mmol/L) at 30 °C, total bacterial protein was extracted and detected in SDS‐PAGE. The expressed protein, fused with GST‐tag, was purified by affinity chromatography over a glutathione‐Sepharose 4B column.

Monospecific polyclonal antiserum against the recombinant *Aloc*SWP2 or GST was produced from rabbits using a standard shortened immunization protocol, conducted by a commercial facility (Vital River Laboratory Animal Technology Co., Ltd., Beijing, China). A rabbit was immunized via intradermal injection at the dorsum with 0.25 g of GST‐*Aloc*SWP2 fusion protein mixed with Freund's adjuvant (1:1[v/v]), followed by booster injections with incomplete Freund's adjuvant (1:1[v/v]) every 2 wks. Sera were collected preimmunization 2 wks after the third injection and stored at −80 °C.

GST‐*Aloc*SWP2 fusion protein and *A. locustae* “SDS extract” samples were subjected to SDS‐PAGE on 12% polyacrylamide gels. After electrophoresis, proteins were strained with CBB and transferred onto nitrocellulose filter membrane (Whatman, 10401196; Dassel, Germany) for western blot analysis. The membranes were blocked with 10 ml with 5%(w/v) nonfat milk in PBST (0.05% [v/v] Tween‐20 in PBS) (blocking solution) for 2 h, and then incubated at room temperature for 1 h with 10 μg/ml rabbit anti‐*Aloc*SWP2 antibody in PBST. Membranes were washed thrice in PBST; the membranes were then incubated at room temperature for 1 h with goat anti‐rabbit IgG conjugated with HRP (1:10,000) as second antibody in PBST with 5%(w/v) nonfat milk, and then finally washed with PBST, and reactivity was detected using enhanced chemiluminescence (ECL) reagent (thermo) in a Western blot analysis by FluorChemM system (ProteinSimple, San Jose, CA).

### Immunofluorescence assay

A host cell binding assay was designed to determine which Spore wall protein interacted with the host cell surface according to Southern et al. (2007). The purified spores (5 × 10^8^) were washed with PBS and fixed with 80% cold acetone for 20 min at room temperature. Slides of fixed spores were permeabilized by PBS with 0.5% Triton X‐100 for 15 min. After washing with PBS with 0.05% Tween‐20 for three times, the samples were blocked in 5% nonfat milk for 30 min, followed by incubation with anti‐*Aloc*SWP2 antibody, diluted at 10 μg/ml in PBS with 0.1% Triton X‐100, at 37 °C for 1.5 h. Following a second wash step, the spores were incubated with a 1:64 dilution of FITC‐conjugated goat anti‐rabbit IgG for 1 h. DAPI (4′6‐diamidino‐2‐phenylindole) (5 μg/ml) was used to stain DNA for 30 min at room temperature before the final wash. Finally, the slides were mounted with a glycerol solution, and visualized on an OLYMPUS IX81 fluorescence microscope (excitation wavelength of FITC‐IgG: 495 nm; DAPI: 359 nm) (Accoceberry et al. 1999; Alfa Cisse et al. 2002).

### Immunochemistry localization

Transmission electron microscopy immunolabeling (IEM) experiments were conducted with *A. locustae*‐infected. The chemical fixation was done via immersion of locust fat bodies into a mixture of paraformaldehyde (4%) and glutaraldehyde (2%) in 0.1 M PBS (pH 7.4), followed by dehydration in an ethanol series. The samples were embedded in LR White resin (Taab, Aldermaston, Berks, U.K.) via polymerization at 60 °C in tightly closed gelatin capsules. Ultrathin sections were cut with a glass knife on ultramicrotome and mounted on Formvar‐coated grids.

For immunocytochemistry, the grids were subsequently floated on 30 μl droplets of the following solutions, mainly adapted from Steinbrecht (Steinbrecht 1992, 1993): in brief, PBS containing 50 mM glycine, PBGT (PBS containing 0.2% gelatin, 1% bovine serum albumin, and 0.02% Tween‐20), primary antibody diluted with PBGT, six washings with PBGT, secondary antibody in PBGT, two washings on each PBGT, PBS glycine, PBS, and water. Optional silver intensification (Danscher 1981) increased the size of the gold granules from 10 to about 40 nm; 2% uranyl acetate increased the tissue contrast for observation in transmission electron microscope (HITACHI H‐7800; Tokyo, Japan).

Immunocytochemical labeling was done on sections of the fat bodies of three male and three female adult infected locusts. The following antibodies were used in this study: anti‐GST‐*Aloc*SWP2 antibody and anti‐GST antibody. The primary antibodies were diluted at 10 μg/ml in PBGT and incubated at 4 °C overnight. As a control, the primary antiserum was replaced by serum from a healthy rabbit. The secondary antibody was anti‐rabbit IgG, coupled to 10 nm colloidal gold (AuroProbe™ EM, GAR G10, Amersham, U.K.), diluted 1:20 and incubated at room temperature for 60–120 min.

### RT‐PCR analysis

Two or three days after the 3rd instar nymph locusts, which were starvation treatments for 4 h, they were fed with 10^7^ spores of *A. locustae* coated on 20 mm × 5 mm corn leaves and were used to detect when *A. locustae* entered the fat body and propagated. Locusts’ fat bodies from the 1st day to the 19th day since inoculation were collected every 2 days. Based on DNA sequences, primers were designed as follows for RT‐PCR on the transcription of the *AlocSWP2* gene and the locust *actin* gene (*LmigActin*) (GenBank accession number: KC118986.1). *AlocSWP2*‐F: 5′‐ATGAACGGGATTATTCTTAGCG‐3′; *AlocSWP2*‐R: 5′‐TTAAGCAGAGTAGAAGCAGCG‐3′. *LmigActin*‐F: 5′‐GCAAAGCTGGCTTCGCCG‐3′; *LmigActin*‐R: 5′‐ATGTTCCTCGGGCGCCAC‐3′. PCR reactions were performed under the following thermal program: 94 °C for 10 min; 30 cycles of 94 °C for 15 s, 58 °C for 15 s, 72 °C for 30 s; followed by one cycle at 72 °C for 10 min. PCR products were run on 1.2% agarose gels and visualized via ethidium bromide staining, then observed and photographed.

### Production of dsRNA

To specifically reduce *Aloc*SWP2 protein levels in *A. locustae*, we used RNAi to knockdown the *Aloc*SWP2 expression. Primers for the full *AlocSWP2* gene, without the signal coding sequence used were: *AlocSWP2*‐F: 5′‐TAATACGACTCACTATAGGATCAGAACGGCAGCGACA‐3′; *AlocSWP2*‐R: 5′‐TAATACGACTCACTATAGGAGCAGAGTAGAAGCAGCGGT‐3′. Templates for dsRNA preparation were PCR‐derived fragments amplified between two T7 promoter sequences (underlined). The double‐stranded RNA fragments were synthesized using T7 RiboMAX™ Express RNAi System (Promega, Madison, WI). A green fluorescent protein (GFP)‐derived dsRNA (dsRNA‐GFP) has been used as exogenous control (Nunes et al. 2013). Finally, each locust infected with *A. locustae* was injected with 10 μg of the dsRNA or buffer.

### Semi‐quantitative PCR and quantitative reverse transcription PCR analysis

To observe the effect of RNAi treatment on *AlocSWP2* transcription, semi‐quantitative PCR and qRT‐PCR experiments were performed according to the manufacturer protocol. On the 10th day after *A. locustae* infection, when the spores just enter into fat body to express *Aloc*SWP2, dsRNA was injected into the locust. Three days later, the mRNA was extracted from dissected tissues using Trizol. The cDNA was synthesized from 1 mg of total RNA with MLV reverse II RT‐PCR system (Promega). Both *AlocSWP2* and *A. locustae actin* sequences were amplified from the same cDNA (3–5 individuals per replicate). The *actin* sequence of *A. locustae* (GenBank accession number: AF031702.1) was used as an internal control, with the primers *AlocActin*‐F: 5′‐GGCATTCCCAAGCACAAAGG‐3′; *AlocActin*‐R: 5′‐ACAGAACAGCCTGAATCGCA‐3′. The mRNA from the *AlocSWP2* gene was amplified using the *AlocSWP2*‐F/R primers. Semi‐quantitative PCR was performed via MyCycler (Bio‐Rad), qRT‐PCR was performed using a LightCycler Nano (Roche, Basel, Switzerland), and the specificity of amplification was confirmed through melting curve analysis. Gene transcription difference was calculated by the 2^−ΔΔCt^ values method (Livak and Schmittgen 2001). For normalization between samples, the mRNA levels from the *actin* of *A. locustae* gene were validated experimentally for each generation and treatment, with the geometric mean then used for normalization according to the strategy described previously (Vandesompele et al. 2002). The PCR program was as following: 94 °C for 10 min, followed by 45 cycles for qRT‐PCR (semi‐quantitative PCR at 25 cycles), each cycle consisting of 94 °C for 15 s and 58 °C for 15 s and 72 °C for 15 s. At the end, samples were incubated to 4 °C for 10 min.

### RNAi of *AlocSWP2* in infected locusts

Approximately 160 locusts were collected and transferred into individual mini‐boxes. According to the previously described method, locusts were infected with *A. locustae*. At the 10th day postinfection, dsRNA was injected into the locust enterocelia lymph system to knockdown *A. locustae* of *AlocSWP2* associated with the locust. Synthesis of dsRNA was essentially conducted as described (Maori et al. 2009; Paldi et al. 2010). Dead locusts, from natural and accidental causes within 10 d, as well as from failed dsRNA injections were discarded. Then, locusts were fed and assessed for mortality every day. Samples of dead locusts (larvae and adults) were collected daily from each mini‐box and immediately frozen in −80 °C for further analysis. One group of mini‐boxes was supplemented with *Aloc*SWP2‐specific dsRNA, while two infected control groups were supplemented with dsRNA buffer and GFP‐specific dsRNA. To test dsRNA toxicity, another group of mini‐boxes containing uninfected locusts was provided with *Aloc*SWP2‐specific dsRNA, while other control groups were left completely untreated. The survival curves were compared using Kaplan–Meier and Cox's proportional hazards model for assessing the variables that affect locust survival.

Sixty females of locusts were randomly assigned to three treatment groups for different average spore loads (intensity) in RNAi. The first group was supplemented with *Aloc*SWP2‐specific dsRNA (*Aloc*SWP2 RNAi), while two infected control groups were supplemented with dsRNA buffer (control) and GFP‐specific dsRNA (GFP RNAi), respectively. As previous treatment strategy, locusts were infected with *A. locustae*. At the 10th day postinfection, RNAi treatment was conducted. Samples of dead locusts were collected immediately and frozen in −80 °C, until at the 16th day postinfection, all locusts were obtained for spore load counts with a hemocytometer (Plischuk et al. 2013). All statistical *t*‐tests (and nonparametric tests) followed by two‐tailed comparison tests were performed using GraphPad Prism version 6.00 for Windows, (GraphPad Software Inc., La Jolla, CA).

## Results

### Identification and characterization of a spore wall protein from *Antonospora locustae*

**Figure 1:**
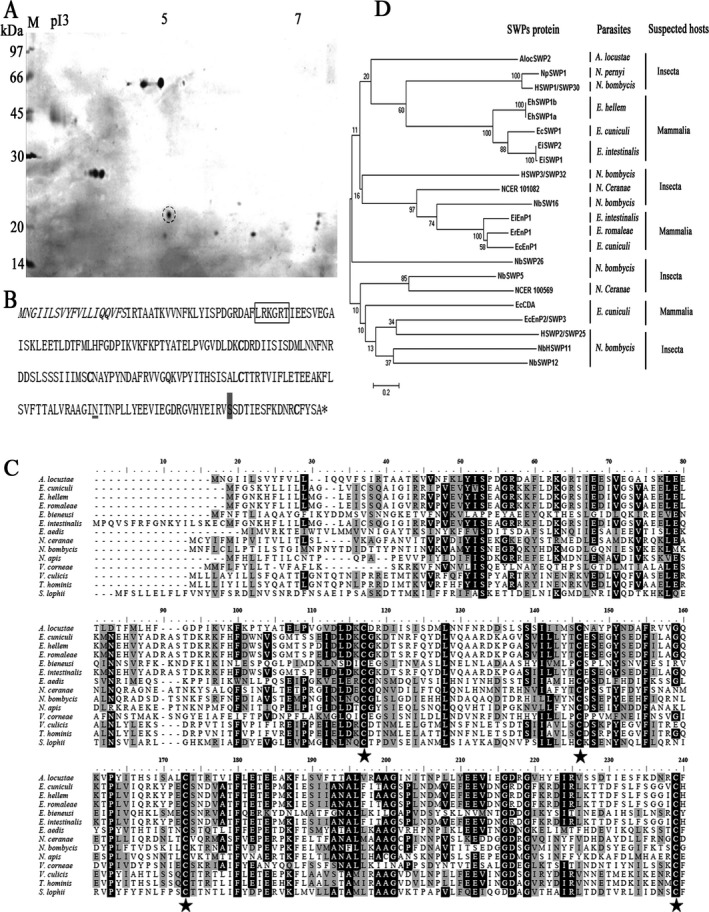
A novel protein, *Aloc* SWP2, isolated from *Antonospora locustae* spores. (**A**) Proteins profile extracted from *A. locustae* spores in 2 dimensions electrophoresis SDS PAGE. The target protein highlighted (dashed circle). M. protein molecular weight markers, pI, isoelectric point. (**B**) Amino acid sequence of *Aloc* SWP2, showing the signal peptide (italicized), and the predicted HBM (box), the asparagine predicted to be N‐glycosylated (underlined) and the potential GPI‐modification (grey shade). (**C**) Sequence alignment of the amino sequence of *Aloc* SWP2 with other 14 amino acid sequences of putative spore wall proteins of other species in Microsporidia. Identical and similar residues are highlighted in black and grey, respectively. The conservative cysteine sequences of these Microsporidia marked with an asterisk. Microsporidia and UniProt database ID:* A. locustae* (Q6E6F1); *Encephalitozoon cuniculi* (Q8SWG8); *Encephalitozoon hellem* (I6UB54); *Encephalitozoon romaleae* (I6ZS97); *Enterocytozoon bieneusi* (B7XHL8); *Encephalitozoon intestinalis* (E0S5M3); *Edhazardia aedis* (J9DKY7); *Nosema ceranae* (C4VBU8); *Nosema bombycis* (R0KVG4); *Nosema apis* (T0KWN0); *Vittaforma corneae* (L2GPT0); *Vavraia culicis* (L2GWD6); *Trachipleistophora hominis* (L7JV52); *Spraguea lophii* (S7W7E5). (**D**) Molecular phylogenetic analysis of spore wall protein sequences from Microsporidia. The evolutionary history was inferred via the Maximum Likelihood method based on the JTT matrix‐based model and conducted in MEGA6 (Tamura et al. 2013). The bootstrap consensus tree inferred from 1,000 replicates is taken to represent the evolutionary history of the taxa analyzed.

**Figure 2:**
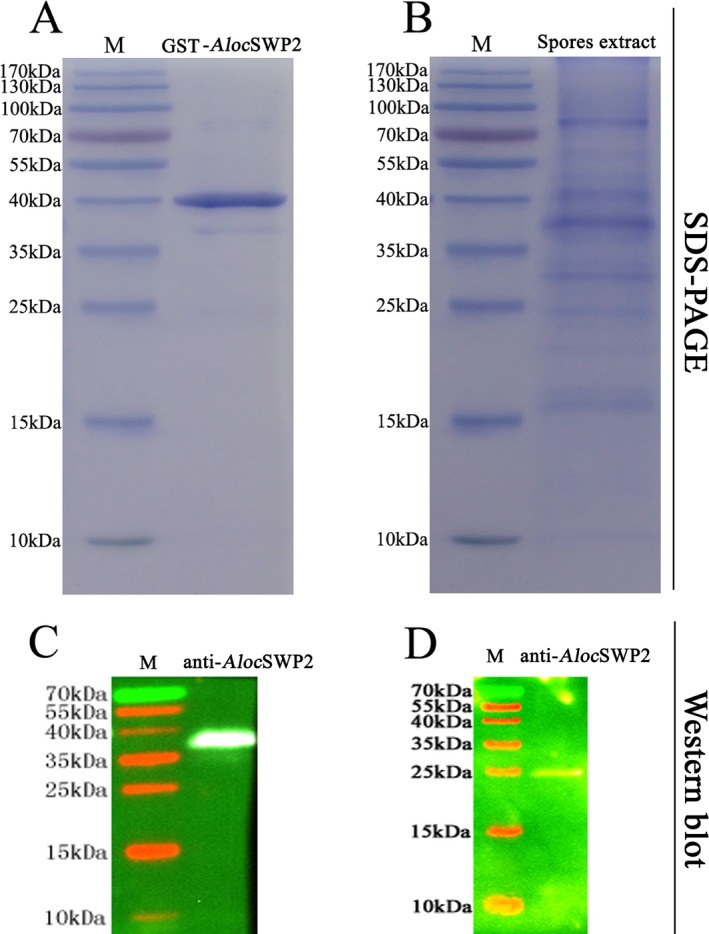
SDS‐PAGE and Western blot analysis of *Aloc* SWP2. (**A**) SDS‐PAGE of purified recombinant *Aloc* SWP2. (**B**) SDS‐PAGE of the SDS extract of spore proteins from *Antonospora locustae*. (**C**) Western blot of purified recombinant *Aloc* SWP2, which contains a GST label, using an anti‐*Aloc* SWP2 antibody. (**D**) Western blot of extract of endogenous spore proteins using an anti‐*Aloc* SWP2 antibody. M, protein molecular weight marker.

About 20 protein spots were separated by 2‐DE of the protein preparation extracted from *A. locustae* spore walls (Fig. 1A). A spore wall protein, with an approximate molecular weight and pI of 20 kDa and 5, respectively was extracted and digested by trypsin, and the protein sequence identified by peptide fingerprinting with MALDI‐TOF MS and expressed protein sequence comparisons, as *AlocSWP2* (Fig. 1B). RACE‐PCR was then used to obtain the full‐length gene (GenBank with accession number KX255658), with an open reading frame of 669 bp encoding for a 222‐amino acid protein, with a predicted molecular mass of 25 kDa and pI of 5.16, consisting of a 203 amino acid mature protein and a 19 amino acid signal peptide. The predicted mass and pI were consistent with those observed for the protein isolated by 2‐DE. The protein contains a potential GPI‐modification site in the C‐terminal (Ser, 206 amino acids site) and a heparin‐binding motif (HBM) composed of LRKGRT (amino acids 43–48), which conforms to the consensus sequence for HBMs, “XBBXBX”. Most interestingly, there are four conserved cysteines (Fig. 1C, asterisks), located at 117, 146, 173, and 23, as well as several other conserved amino acids in these proteins, such as Leucine (128), Phenylalanine (155, 180), Proline (163), and glycine (119) although the proteins are from different microsporidian parasites whose hosts vary from Insecta to Mammalia. However, the phylogenetic analysis showed that *Aloc*SWP2 fall into a specific single branch that is quite different from other proteins, both from insects and mammals in the phylogenetic tree (Fig. 1D).The purified recombinant protein (Fig. 2A) and the protein containing extract from *A. locustae* (Fig. 2B) were analysed by 12% SDS‐PAGE followed by western blotting. The anti‐GST‐*Aloc*SWP2 polyclonal antibody labeled the recombinant GST‐fused protein that showed a molecular weight of ~40 kDa, consistent to the calculated molecular weight of the fusion protein (GST‐*Aloc*SWP2 without the signal peptide; Fig. 2C). A single ~25 kDa protein band was detected by anti‐GST‐*Aloc*SWP2 polyclonal antibody in SDS extract (Fig. 2D). Preimmune serum was used as a negative control in all Western blots (data not shown) and no nonspecific bands were detected. This proved that a polyclonal antibody was successfully produced in rabbits and had a strong activity against purified recombinant *Aloc*SWP2 or endogenous *Aloc*SWP2 in *A. locustae*.

### *Aloc*SWP2 was localized in the spore wall

**Figure 3:**
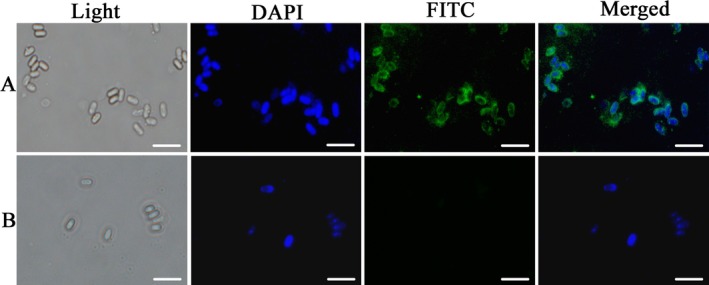
Localization of *Aloc* SWP2 by immunofluorescence assay. Light, purified *Antonospora locustae* were observed via light microscope only. (**A**) Blue fluorescence signals show samples stained with DAPI, which were observed with a fluorescence microscope; green fluorescence signals show the samples treated with anti‐*Aloc* SWP2 antibody and FITC‐conjugated goat anti‐rabbit IgG. (**B**) Negative control. The anti‐*Aloc* SWP2 antibody was diluted at 10 μg/ml in blocking solution. The secondary antibody was FITC‐conjugated goat anti‐rabbit IgG was diluted 1:64. Bar, 10 μm.

*Aloc*SWP2 was detected in the wall of mature spores via indirect immunofluorescence assay (IFA) (Fig. 3). *Antonospora locustae* spores were treated with the anti‐GST‐*Aloc*SWP2 antibody, which labeled the spore wall (green fluorescent signal), while DAPI stained DNA was visible within the spores (Fig. 3A). No background green fluorescence was observed in spores treated with anti‐GST (Fig. 3B). The results suggest that *Aloc*SWP2 was localized on the surface of the spore coat.

**Figure 4:**
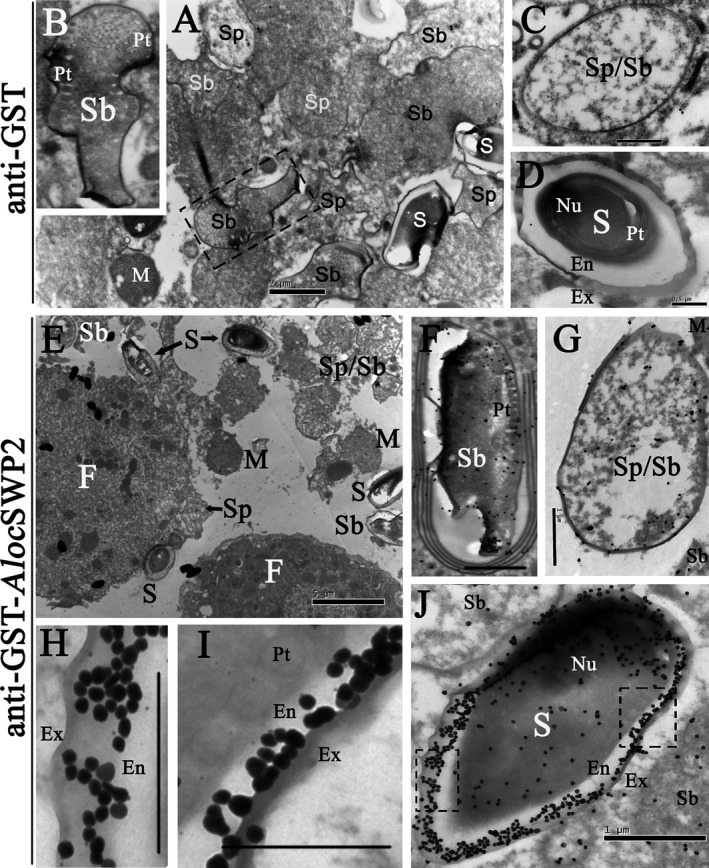
Immunocytochemistry localization of *Aloc* SWP2 in fat bodies of locusts. (**A–D**) Negative control, *A. locustae‐*infected locust fat cells treated with anti‐GST antibody. (**E–K**) Sections of *A. locustae‐*infected locust fat cells incubated with anti‐GST‐ *Aloc* SWP2 antibody. Ex = exospore; En = endospore; Pt = polar tube; Nu = nucleus; Sp = sporont; Sb = sporoblast; M = meront; S = mature spores; F = locust fat cell. Bars in D, H, I, 0.5 μm; Bars in A, C, F, G, J, 1 μm; Bar in B, 2 μm; Bar in E, 5 μm.

Furthermore, the IEM experiments were used to further confirm that *Aloc*SWP2 was a spore wall protein. The fat body of the host locust was used to provide more detail about the protein in spore development process. Dark granules in ultra‐sections are anti‐GST‐*Aloc*SWP2 antibody coupled to gold particles, and then amplified by silver staining, were observed in both the endospore and exospores wall, indicating that this protein is expressed in both (Fig. 4). The dark granules of anti‐*Aloc*SWP2 were also observed in the spore walls of the sporoblast and sporont, but much less intense than in mature spores, indicating that the protein expression begins with spore wall formation. We found that the anti‐GST‐*Aloc*SWP2 antibody labeled the layers of spore wall during formation (Fig. 4E–G), and the thickness of this label increased with thickness of spore wall (Fig. 4F–J). After maturation, *Aloc*SWP2 is mainly localized to the endospore (Fig. 4J), shows in magnified pictures (Fig. 4H and I). Notably, dark granules distributed in both sporoblast and sporont (Fig. 4F and G) in disarray; however, they were arranged in an orderly fashion in the mature spore (Fig. 4J). *Aloc*SWP2 is differently distributed during the life cycle stages of the sporoblasts and mature spores of *A. locustae*. No labeling was observed in the negative controls labeled with anti‐GST (Fig. 4A–D).

### RNAi of *Aloc*SWP2 reduced the mortality of locusts infected by *Antonospora locustae* through its sporulation

**Figure 5:**
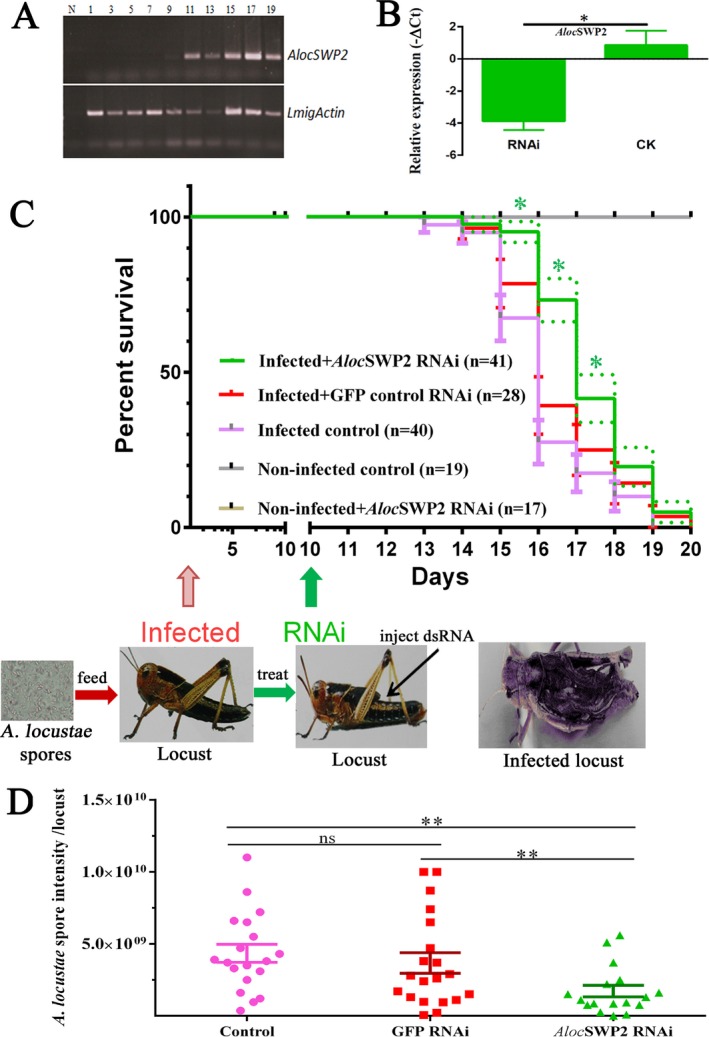
Reduction of the mortality of locusts infected by *Antonospora locustae* after depression of *Aloc* SWP2 via RNAi. (**A**) *AlocSWP2* gene expression was detected via RT‐PCR in the fat body of locusts after inoculation. *LmigActin* is a locust *actin* gene. Lane: 1, 3, …19 locusts on the days after *A. locustae* inoculation, N is the negative control. (**B**) qRT‐PCR experimental result of micro‐injection with dsRNA of *AlocSWP2* to interfere with the expression of the gene. Reference gene is *A. locustae actin*. Bar height denotes the mean average of sample‐specific −ΔCt values, and values are plotted as means ± SEM from at least three repeats. Significant difference at the 0.0016 level. (**C**) The survivals of locusts inoculated with *A. locustae* spores or non‐inoculated*,* and those after treatment with RNAi of *AlocSWP2*. The 3rd instar nymphs of the locust were infected by *A. locustae*, then treated with or without RNAi of *AlocSWP2*. The number (*n*) of in each group treatment group is noted and three independent repeats of each experiment were done. The arrow indicates the time (days) postinoculation when injection was performed. The Kaplan–Meier method in Graphpad Prism 6 was used to analyze survival data. The *t*‐test was used for significance analysis, the *P* value of survival proportions of Infected + *Aloc* SWP2 RNAi group against Infected + GFP control RNAi group by paired one tail *t*‐test was 0.0278, which was significantly different. The *P* value of survival proportions of Infected + *Aloc* SWP2 RNAi group against Infected control group by paired one tail *t*‐test was 0.0209, which was significantly different. Bars and dotted line height denote the mean average of SEM. (**D**) Comparisons of *A. locustae* spores per locust between the treatments (average spore load). *P <* *0.05, **P <* *0.01.

In order to determine when *AlocSWP2* starts to be expressed in its host, RT‐PCR of *AlocSWP2* gene was performed after inoculation (Fig. 5A), showing no expression until 9–11 d postinoculation. For RNAi treatment, dsRNA was prepared and injected into the locust hemolymph on the 10^th^ day postinoculation with spores of *A. locustae*. Total RNA was exacted 3 days later for semi‐quantitative PCR and qRT‐PCR experiments. *AlocSWP2* transcription was reduced in both detection of RNAi by semi‐quantitative PCR (Fig. S1) and in the fat body of infected locust after injection of *AlocSWP2* dsRNA was significantly reduced approximately 26 fold compared to those in the mock‐injected group (no RNAi; Fig. 5B and Table S1). This proved that injection with dsRNA of *AlocSWP2* can effectively depress expression of *AlocSWP2*.

To examine the effects of RNAi of *AlocSWP2* on pathogenicity of the parasite to its host, survival rates were measured with and without the RNAi treatment by injection 10 d postinoculation with *A. locustae* spores. Mortality rates from five treatment groups were compared: locusts infected with *A. locustae* spores and treated with RNAi against *AlocSWP2* (infected + *AlocSWP2* RNAi); infected locust without RNAi against *AlocSWP2* (infected control) and Infected + GFP control RNAi group; healthy locust (noninfected control); and healthy locusts injected with dsRNA against *AlocSWP2* (noninfected +* AlocSWP2* RNAi). Both survival proportions of Infected + *Aloc*SWP2 RNAi group against Infected + GFP control RNAi group and the group of Infected control was significantly different. No deaths occurred during the examined period, exceptions happened due to natural and accidental causes within 10 days, as well as due to failed dsRNA injections. However, the mortalities of the infected locusts treated with RNAi against *AlocSWP2 mRNA* were significance lower on the 15th, 16th, and 17th days after inoculation with parasite spores than those of locusts in infected control group (Fig. 5C and Table S2).

The effects of treatment on *A. locustae* intensity (average spore load) are summarized in Fig. 5D. Among the *Aloc*SWP2 RNAi treatments that received spore inoculations, significant differences were found in spore intensities (*P *<* *0.01). There were no significant differences in dsRNA buffer (Control) and GFP‐specific dsRNA (GFP RNAi) treatments (*P *=* *0.6131). After silencing *Aloc*SWP2, *A. locustae* failed to produce spores; consequently, the host suffered less from the parasite burden and demonstrated reduced mortality in the case of RNAi treatments.

## Discussion

Some spore wall proteins have been identified since the late 1990s. The genes and proteins of microsporidians can now be identified rapidly since the genomes of several microsporidians have been sequenced, such as *E. cuniculi, N. bombycis*. So far, only about 20 spore wall proteins have been reported both in the *Encephalitozoonidae* family and in *N. bombycis*. Typical spore wall proteins of microsporidian may have the following features (Table 1): (i) 15–20 amino acid signal peptide sequences; a predicted GPI‐anchor site indicating that it is a membrane or spore wall protein; glycosylation and/or other posttranslational modification; glycine or serine rich; and have adherence domains such as HBM. (ii) These proteins are generally cysteine rich, with most spore wall proteins containing four conservative cysteine required disulfide bond formation, however, there is one such protein without any cysteines. (iii) The sequences of SWPs are highly diverse. In the same host, the sequences of amino acid of spore wall proteins may be similar, for example SWP25 and SWP26 in *N. bombycis*. However, no homologous proteins were found in *E. cuniculi* so far (Hayman et al. 2001; Li et al. 2009; Wu et al. 2009). In our study, four conserved cysteines were identified in *Aloc*SWP2 and other Microsporidia, which infect both insects and mammals. Furthermore, this feature may lead to them resembling tertiary structures or functions via disulfide‐bonded linkages. Bohne et al. studied the spore wall protein *E. cuniculi*, and found that the cysteines and N‐terminal signal peptide sequences were conserved, indicating similar tertiary structure or function (Bohne et al. 2000).

**Table 1:** The identified spore wall proteins of Microsporidia

Protein	Source	Mw (kDa)	Full length (nt)/amino acids (aa)	PI	Signal peptide/length (aa)	Phosphorylation/N‐glycosylation/O‐glycosylation	Subcellular localization	Functional domain	References/GenBank ID
EiSWP1	*Encephalitozoon intestinalis*	41.5	1167/388	4.78	Yes/18	36/1/37	External wall	–	Hayman et al. (2001); AF355750.1
EiSWP2	*E. intestinalis*	107.2	3009/1002	3.68	Yes/18	83/1/1	External wall	–	Hayman et al. (2001); AF355749.1
EiEnP1	*E. intestinalis*	39.1	1047/348	8.84	Yes/16	28/3/1	Internal wall/external wall/polar membrane layer	HBM	Corradi et al. (2010) and Pombert et al. (2012); XM_003072248
ErEnP1	*E. romaleae*	39.5	1047/348	9.12	Yes/16	15/3/3	–	HBM	Pombert et al. (2012); XM_009265627.1
EcSWP1	*Encephalitozoon cuniculi*	45.9	1353/450	4.96	Yes/18	66/0/52	External wall	–	Katinka et al. (2001); NM_001042116.1
EcCDA	*E. cuniculi*	28.1	765/254	4.43	Yes/15	16/0/0	Endospore plasma membrane	Glycoside hydrolase/Deacetylase	Brosson et al. (2005); NC_003237.1
EcEnP1	*E. cuniculi*	40.6	1074/357	9.07	Yes/16	20/1/3	Endospore	HBM	Katinka et al. (2001) and Peuvel‐Fanget et al. (2006); XM_960824
EcEnP2/SWP3	*E. cuniculi*	22.5	666/221	8.42	Yes/20	31/0/27	Endospore/plasmalemma	Transmembrane	Katinka et al. (2001), Peuvel‐Fanget et al. (2006), and Xu et al. (2006); NC_003242.2
EhSWP1a	*Encephalitozoon hellem*	54.9	1530/509	4.30	Yes/18	38/3/27	Extracellular spores	–	Polonais et al. (2010); FJ870923
EhSWP1b	*E. hellem*	57.9	1602/533	4.64	Yes/18	44/3/29	Extracellular spores	–	Polonais et al. (2010); FJ870924
NbSW16	*Nosema bombycis*	22.5	666/221	8.42	Yes/15	34/2/29	Exospore	HBM	Wang et al. (2015); KB908937.1
NbHSWP11	*N. bombycis*	52.3	1341/446	9.27	No	32/0/1	Internal wall/external wall	DnaJ domain	Yang et al. (2014); EF683111
SWP5	*N. bombycis*	20.3	561/186	4.39	Yes/22	13/0/6	Exospore/polar tube	–	Li et al. (2012); HQ881497
NbSWP12	*N. bombycis*	26.6	687/228	6.78	No	13/1/0	Internal wall/external wall	BAR‐2 domain	Chen et al. (2013); KC193258
SWP25/HSWP2	*N. bombycis*	30.7	807/268	8.45	Yes/25	15/2/6	Endospore	HBM	Wu et al. (2008, 2009); EF683102
SWP26	*N. bombycis*	25.7	672/223	5.09	Yes/16	13/0/0	Endospore/plasma membrane/exospore	HBM	Li et al. (2009); EU677842
SWP30/HSWP1	*N. bombycis*	32.1	837/278	7.95	Yes/19	22/1/4	Endospore	–	Wu et al. (2008); EF683101
SWP32/HSWP3	*N. bombycis*	37.4	951/316	7.29	Yes/18	12/1/0	Exospore	–	Wu et al. (2008); EF683103
NbSWP7	*N. bombycis*	32.3	864/287	4.78	Yes/19	7/0/1	Exospore/endospore	–	Yang et al. (2015); EOB13707.1
NbSWP9	*N. bombycis*	42.8	1104/367	8.32	No	7/0/3	Exospore/endospore/polar tube	Transmembrane helix region	Yang et al. (2015, 2017); EOB13793.1
NCER_100569	*N. ceranae*	20.4	552/183	4.37	Yes/20	8/0/2	–	–	Cornman et al. (2009) and Li et al. (2012); XM_002996306.1
NCER_101082	*N. ceranae*	48.4	1281/426	8.37	Yes/22	30/4/11	–	–	Cornman et al. (2009) and Pelin et al. (2015); XM_002995858 or KKO74775
SWP1	*Nosema pernyi*	32.0	837/278	7.27	Yes/19	18/1/6	–	–	KJ573111
*Aloc*SWP2	*Antonospora locustae*	25.0	669/222	5.16	Yes/19	6/1/0	Internal wall/external wall	HBM	KX255658

It has been reported that Microsporidia adhere to host cells that are dominated by GAGs on surface (Hayman et al. 2005), with some spore wall proteins acting as a ligand during spore adherence to the host (Brosson et al. 2005; Southern et al. 2007). Heparin‐binding motifs (HBMs) also exist in the amino acid sequence of *Aloc*SWP2. It was suggested that HBMs interact with extracellular GAGs, with the consensus sequence necessary for protein‐heparin interaction characterized by “XBBXBX,” “XBBBXXBX”, or “XBBXBXBBX” (“X” is any neutral amino acid and “B” is a positively charged basic amino acid) (Wu et al. 2008). Typical spore wall proteins from Microsporidia have features that include HBMs (Table 1), with one such motif identified in *Aloc*SWP2. Recent reports showed that blocking either SWP16 or SWP11 using in vitro antibody treatment caused a 20% decrease in the adherence of *N. bombycis* spores to host cells for either case (Wang et al. 2015; Yang et al. 2014). By using a host cell binding assay, recombinant SWP26 protein (with HBM) can inhibit *N. bombycis* adherence by 10%, resulting in decreased host cell infection. In contrast, mutant rSWP26 (without HBM) did not inhibit spore adherence (Li et al. 2009). The HBMs may be important for adherence of the spore wall to host cells during the passage of the spores through the gastrointestinal tract, facilitating invasion.

In our research, *AlocSWP2* transcription in the fat body of infected locusts by injection of *AlocSWP2*‐specific dsRNA was significantly reduced as detected by qRT‐PCR. In fact, RNAi were demonstrated to be efficiently used to regulate microsporidian gene expression in honeybee or silkworm hosts (Paldi et al. 2010; Pan et al. 2017). Survival percentages of locusts that were infected with *A. locustae* without RNAi of *AlocSWP2* were lower than those of the infected locusts that were injected with dsRNA of *AlocSWP2* gene, suggesting that this protein may be involved in host pathogenicity. Further analysis revealed that the amount of spores of RNAi of *AlocSWP2* in each infected locust was much lesser than those of infected locusts without RNAi of the gene. Therefore, considering the interference of RNA within host fat body, less expression of the protein caused fewer amount of sporonts and spores, resulting in lower host mortality. As we know, the pathogenicity of microsporidian mainly depends on the amount of spores, which cause an acute anaphylactic reaction (Selman 1982; Szumowski and Troemel 2015). Interestingly, here we found that locusts in the RNAi treatments specifically knockdown certain structural protein such as *Aloc*SWP2, and consequently had lower *A. locustae* intensities, and higher survival. We further speculate that the expression of some of the spore wall proteins of microsporidian may contribute to the sporulation of spores or other functions, which influence the pathogenicity of this microporidian to its host. This is the first demonstration that a spore wall protein from *A. locustae* contributes to the mortality of its host, and provides a novel spore wall protein of Miscroporidia whose hosts can be found within both Insecta and Mammalia contributing to sporulation.

## Supporting information


**Figure S1.** The results of semi‐quantitative PCR for the detection of RNAi of *AlocSWP2*.
**Table S1.** Numerical data of qRT‐PCR to check the results of RNAi of *AlocSWP2*.
**Table S2.** Raw data of the survival assay after RNAi of *AlocSWP2*.
