# Supplementary material for: A Novel Spore Wall Protein from Antonospora locustae (Microsporidia: Nosematidae) Contributes to Sporulation
Source: J Eukaryot Microbiol. 2017 Apr 10;64(6):779–91. doi: 10.1111/jeu.12410 (PMC5697631; doi:10.1111/jeu.12410)
Supplement: Supplementary file 1 — Figure S1. The results of semi‐quantitative PCR for the detection of RNAi of AlocSWP2. Table S1. Numerical data of qRT‐PCR to check the results of RNAi of AlocSWP2. Table S2. Raw data of the survival assay after RNAi of AlocSWP2. [file JEU-64-779-s001.pdf]

SUPPORTING INFORMATION

A Novel Spore Wall Protein from *Antonospora locustae* (Microsporidia: Nosematidae) Contributes to Sporulation by Longxin Chen, Runting Li, Yinwei You, Kun Zhang, Long Zhang

Fig. S1. The results of semi-quantitative PCR for the detection of RNAi of *AlocSWP2*. M: DNA molecular weight marker. 1: *A. locustae actin*; 2: RNAi of *A. locustae actin*; 3: *AlocSWP2*; 4: RNAi of *AlocSWP2*.
Table S1. Numerical data of qRT-PCR to check the results of RNAi of *AlocSWP2*.
Table S2. Raw data of the survival assay after RNAi of *AlocSWP2*.

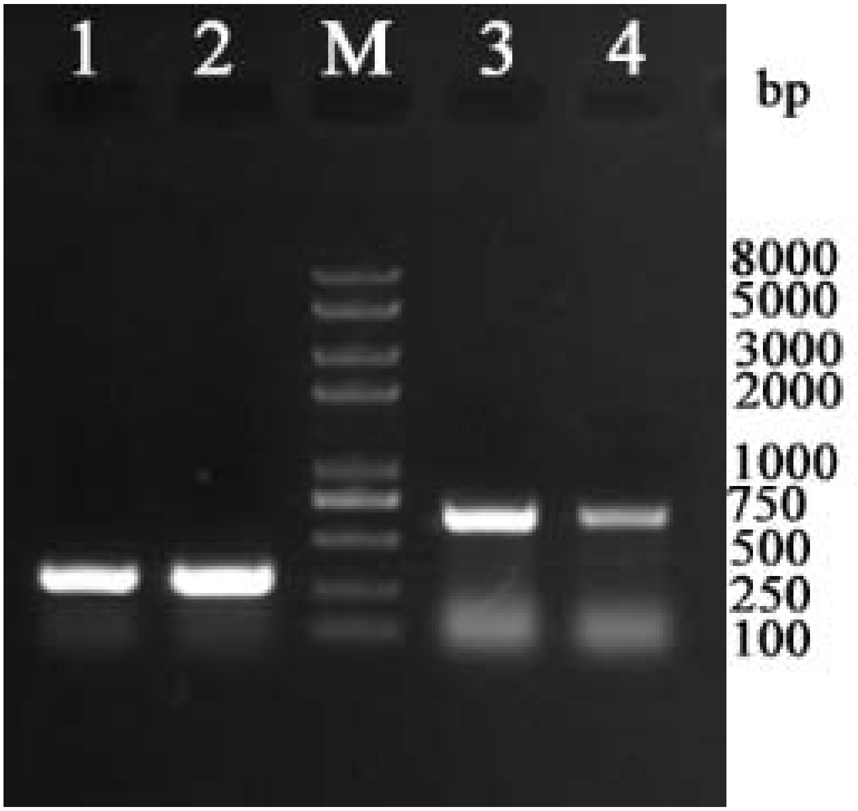

Table S1. Numerical data of qRT-PCR to check the results of RNAi of *Aloc*SWP2.

Sample	Ct（*AlocSWP2*）	Ct（*actin*）	Ct（*AlocSWP2-actin*）	Ct mean（*AlocSWP2*）	Ct SEM（*AlocSWP2*）	Ct mean（*actin*）	Ct SEM（*actin*）	△Ct mean（*AlocSWP2-actin*）	△Ct SEM（*AlocSWP2-actin*）	$2^{-\triangle\triangle Ct}$ mean
**RNAi**	24.567	20.494	3.872	24.559	0.021	20.695	0.553	3.8637	0.574	26.100
	24.575	21.321	3.880							
	24.536	20.271	3.841							
**CK**	19.995	21.2	-1.12	20.276	0.840	21.118	0.077	-0.8423	0.916	
	19.613	21.107	-1.51							
	21.22	21.048	0.102							

Table S2. Raw data of the survival assay after RNAi of *Aloc*SWP2.

Days	Infected+AlocSWP2 RNAi (n=41)	Infected control (n=40)	Non-infected control (n=19)	Non-infected+AlocSWP2 RNAi (n=17)	Infected+GFP control RNAi (n=28)
9					
10					
11					
12					
13					
14	1				
15	1				
16	1				
16	1				
16	1				
16	1				
16	1				
16	1				
16	1				
16	1				
16	1				
17	1				
17	1				
17	1				
17	1				
17	1				
17	1				
17	1				
17	1				
17	1				

17	1	
17	1	
17	1	
17	1	
18	1	
18	1	
18	1	
18	1	
18	1	
18	1	
18	1	
18	1	
18	1	
19	1	
19	1	
19	1	
19	1	
19	1	
19	1	
20	1	
20	0	
20		
9		
10		
11		
12		
13		1
14		1

15	1
15	1
15	1
15	1
15	1
15	1
15	1
15	1
15	1
15	1
15	1
16	1
16	1
16	1
16	1
16	1
16	1
16	1
16	1
16	1
16	1
16	1
16	1
16	1
16	1
16	1
16	1
17	1

17	1	
17	1	
17	1	
18	1	
18	1	
18	1	
19	1	
19	1	
19	1	
19	1	
20		
9		0
10		0
11		0
12		0
13		0
14		0
15		0
16		0
17		0
18		0
19		0
20		0
20		0
20		0
20		0
20		0
20		0

9	0	
10	0	
11	0	
12	0	
13	0	
14	0	
15	0	
16	0	
17	0	
18	0	
19	0	
19	0	
19	0	
19	0	
19	0	
19	0	
19	0	
20	0	
20	0	
9		
10		
11		
12		
13		
14		1
15		1
15		1
15		1

15	1
15	1
16	1
16	1
16	1
16	1
16	1
16	1
16	1
16	1
16	1
16	1
16	1
17	1
17	1
17	1
17	1
18	1
18	1
18	1
19	1
19	1
19	1
20	1
